# 老年肺癌患者的免疫治疗疗效分析

**DOI:** 10.3779/j.issn.1009-3419.2022.102.16

**Published:** 2022-06-20

**Authors:** 孟军 俞, 翔 高, 思芸 付, 卉 张, 娜 秦, 学峰 郝, 仁敬 金, 腾 马, 敬慧 王

**Affiliations:** 1 101149 北京，北京市结核病胸部肿瘤研究所，首都医科大学附属北京胸科医院肿瘤内科 Department of Medical Oncology, Beijing Tuberculosis and Thoracic Tumor Research Institute, Beijing Chest Hospital, Capital Medical University, Beijing 101149, China; 2 101149 北京，北京市结核病胸部肿瘤研究所，首都医科大学附属北京胸科医院肿瘤研究中心 Cancer Research Center, Beijing Tuberculosis and Thoracic Tumor Research Institute, Beijing Chest Hospital, Capital Medical University, Beijing 101149, China

**Keywords:** 肺肿瘤, 老年患者, 免疫治疗, 预后因素, 安全性, Lung neoplasms, Elderly patient, Immunotherapy, Prognostic factor, Safety

## Abstract

**背景与目的:**

以免疫检查点抑制剂(immune checkpoint inhibitors, ICIs)为代表的免疫疗法成为驱动基因阴性晚期非小细胞肺癌(non-small cell lung cancer, NSCLC)的标准治疗方式。但是，肺癌高发于老年患者，而这部分患者很少被纳入重要的临床试验研究。我们旨在研究“真实世界”老年肺癌人群免疫治疗的疗效和安全性。

**方法:**

回顾性分析2018年7月-2021年10月期间接受免疫治疗的老年NSCLC患者和同期的年轻患者，比较不同年龄分组(< 60岁组为中青年组，60岁-74岁为年轻老年组，75岁及以上为一般老年组)患者的客观缓解率(objective response rate, ORR)和无进展生存期(progression-free survival, PFS)，并在各年龄亚组中分析不同临床特征对疗效的影响。

**结果:**

共有21例中青年患者、70例年轻老年患者和15例一般老年患者被纳入本次研究，ORR分别为33.3%、52.8%和53.3%，差异无统计学意义(*P*=0.284)；中位PFS分别9.1个月、7.6个月和10.9个月，差异无统计学意义(*P*=0.654)。进一步对各亚组免疫治疗的预测因素进行分析，发现在年轻老年组和中青年组中，一线接受免疫治疗的患者的PFS更长。三组不良反应的发生率差异无统计学意义(*P*>0.05)。

**结论:**

老年患者接受免疫治疗后的有效性和安全性均与年轻患者相近，一线接受免疫治疗的PFS更长，仍需进一步的前瞻性研究来阐明免疫治疗对老年NSCLC患者的影响。

## Abstract (en)

**Background and objective:**

Immunotherapy represented by immune checkpoint inhibitors (ICIs) has become the standard treatment for patients with non-oncogenic advanced non-small cell lung cancer (NSCLC). While lung cancer is most prevalent in elderly patients, these patients are rarely included in pivotal clinical trial studies. We aimed to describe the efficacy and safety of immunotherapy for elderly patients in the "real-world".

**Methods:**

The data of older NSCLC patients and younger patients who received immunotherapy between July 2018 to October 2021 were retrospectively analyzed and the objective response rate (ORR) and progression-free survival (PFS) in different age groups (less than 60 years old was defined as the young group, 60 years-74 years old was the young old group, 75 years old and above was the old old group) were compared. And the impact of different clinical characteristics on treatment response and prognosis were analyzed in each age subgroup.

**Results:**

A total of 21 young patients, 70 young old patients and 15 old old patients were included in this study, with ORR of 33.3%, 52.8% and 53.3%, respectively, without statistically significant difference (*P*=0.284). The median PFS was 9.1 mon, 7.6 mon and 10.9 mon, respectively, without statistically significant difference (*P*=0.654). Further analysis of the predictors of immunotherapy in each subgroup revealed that patients in the young old group and young group who received immunotherapy in the first line had a longer PFS. The difference of the incidence of adverse events was not statistically significant among the three groups (*P*>0.05).

**Conclusion:**

The efficacy and safety of immunotherapy in elderly patients were similar to those in younger patients, and PFS was superior in the first-line immunotherapy. Further prospective studies are still needed to explore predictors of immunotherapy in elderly NSCLC patients.

根据最新发布的2022美国癌症统计数据，预计2022年美国将出现1, 918, 030例新诊断的癌症病例和609, 360例癌症死亡病例，其中每天约有350人死于肺癌，是癌症死亡的主要原因^[1]^。非小细胞肺癌(non-small cell lung cancer, NSCLC)是肺癌的主要病理类型，约占85%，包括腺癌、鳞癌和大细胞癌，晚期NSCLC患者5年生存率仅为5%^[2]^。以免疫检查点抑制剂(immune checkpoint inhibitors, ICIs)为代表的免疫疗法成为晚期NSCLC的标准治疗方式，使得晚期NSCLC患者5年生存率提升至15.5%-23.2%^[3]^。超过一半的NSCLC患者年龄在70岁以上，近10%的患者年龄在80岁及以上，老年肺癌患者的临床治疗更具有挑战性^[4]^。过往的临床试验例如KEYNOTE-024^[5]^、KEYNOTE-042^[6]^、OAK^[7]^、KEYNOTE-189^[8]^等仅在有限亚组中分析表明老年患者(一般定义为年龄65岁及以上)可能获得与年轻患者相同的免疫治疗益处，并具有可接受的毒性反应。

目前的临床试验结果还不能推广到老年晚期NSCLC患者群体。对老年患者的亚组分析是事后进行的，且参加临床试验的老年患者通常比在日常实际中接受治疗的人群更健康，试验结果缺乏真实世界的相关数据论证。因此，本研究通过收集我院同期接受ICIs治疗的老年NSCLC患者和年轻患者的临床资料，比较真实世界中老年患者和年轻患者接受免疫治疗的疗效和毒副作用。

## 1 资料与方法

### 1.1 一般资料

本研究回顾性收集了2018年7月-2021年10月在北京胸科医院接受ICIs治疗的NSCLC患者的临床诊疗资料。纳入标准：①组织病理确诊为NSCLC；②接受ICIs治疗；③不合并严重的其他脏器合并症；④无活动性自身免疫性疾病。排除标准：①合并其他肿瘤或者多原发肺癌；②无可评价的病灶；③缺乏可用于疗效评价和随访的有效信息。

### 1.2 数据采集

从医院电子病历系统中收集并记录患者的临床诊疗资料，包括年龄、性别、吸烟状况、东部肿瘤协作组体能状况评分(Eastern Cooperative Oncology Group performance status, ECOG PS)、病理类型、临床分期、基因突变类型、程序性死亡配体1(programmed cell death ligand 1, PD-L1)表达水平、免疫治疗方式及治疗线数等。疗效评价采用实体瘤反应评估标准1.1版(Response Evaluation Criteria in Solid Tumors, RECIST 1.1)^[9]^。患者通过定期入院、门诊及电话咨询等方式随访，末次随访日期为2022年4月30日。无进展生存期(progression-free survival, PFS)定义为从接受免疫治疗开始至观察到疾病进展或因任何原因死亡的日期。

### 1.3 统计分析

使用SPSS 22.0软件对数据结果进行统计学分析，绘图使用GraphPad Prism 8进行。使用*χ*^2^检验或*Fisher*精确检验进行率的比较，使用*Kaplan*-*Meier*方法进行生存分析，*Log*-*rank*检验进行组间比较，*Cox*回归模型进行多变量分析。*P* < 0.05认为差异有统计学意义。

## 2 结果

### 2.1 患者特征

共有106例患者纳入本次研究。表 1为106例NSCLC患者的基本特征。其中年龄 < 60岁的即中青年组，占19.8%(*n*=21)，中位年龄为54岁(39岁-59岁)；年龄60岁-74岁的即年轻老年组，占66.0%(*n*=70)，中位年龄为68岁(60岁-74岁)；年龄≥75岁的即一般老年组，占14.2%(*n*=15)，中位年龄为78岁(75岁-82岁)。在接受联合治疗分组中，中青年组中免疫联合化疗13例，免疫联合抗血管2例，免疫联合化疗+抗血管3例；在年轻老年组中免疫联合化疗38例，免疫联合抗血管6例，免疫联合化疗+抗血管8例；而在一般老年组免疫联合化疗10例，免疫联合化疗+抗血管1例。三年龄亚组之间的其他特征基本相似。

**表 1:** 106例患者特征[*n* (%)] Characteristics of 106 patients [*n* (%)]

Factor	The young group (*n*=21)	The young old group (*n*=70)	The old old group (*n*=15)	*P*
Median age (year range)	54 (39-57)	68 (60-74)	78 (75-82)	
Gender				0.726
Male	18 (85.7)	54 (77.1)	12 (80.0)	
Female	3 (14.3)	16 (22.9)	3 (20.0)	
Smoking				> 0.999
Never	6 (28.6)	20 (28.6)	4 (26.7)	
Former/Current	15 (71.4)	50 (71.4)	11 (73.3)	
ECOG PS				0.406
0-1	18 (85.7)	66 (94.3)	14 (93.3)	
2	3 (14.3)	4 (5.7)	1 (6.7)	
Histological type				0.443
Squamous cell	8 (38.1)	25 (35.7)	9 (60.0)	
Non-squamous cell	13 (61.9)	45 (64.3)	6 (40.0)	
TNM stage				0.017
IV	17 (81.0)	42 (60.0)	10 (66.7)	
IIIb	4 (19.0)	28 (40.0)	5 (33.3)	
Mutation status				0.178
*EGFR*	7 (33.3)	7 (10.0)	2 (13.3)	
*KRAS*	2 (9.5)	12 (17.1)	2 (13.3)	
Negative	11 (52.4)	46 (65.7)	8 (53.4)	
PD-L1 expression				0.069
TPS≥50%	4 (19.0)	27 (38.6)	5 (33.3)	
1%≤TPS < 50%	7 (33.3)	14 (20.0)	7 (46.7)	
TPS < 1%	10 (47.6)	25 (35.7)	2 (13.3)	
Median cycles of ICIs (range)	5 (1-31)	5 (1-34)	4 (1-8)	0.877
Immunotherapy protocols				0.555
Monotherapy	3 (14.3)	18 (25.7)	4 (26.7)	
Combination therapy	18 (85.7)	52 (54.3)	11 (73.3)	
Number of treatment lines				0.034
Firstline	7 (33.3)	45 (64.3)	10 (66.7)	
Others	14 (66.7)	25 (35.7)	5 (33.3)	
ECOG PS: Eastern Cooperative Oncology Group performance status; TNM: tumor-node-metastasis; EGFR: epidermal growth factor receptor; KRAS: kirsten rat sarcoma viral oncogene; PD-L1: programmed cell death 1 ligand 1; TPS: tumor proportion score; ICIs: immune checkpoint inhibitors.				

### 2.2 不同年龄组的治疗效果

在中青年组、年轻老年组和一般老年组患者中，客观缓解率(objective response rate, ORR)分别为33.3%、52.8%和53.3%，而疾病控制率(disease control rate, DCR)分别为81.0%、84.3%和100.0%(表 2)。三组之间的ORR和DCR差异无统计学意义(*P* > 0.05)。

**表 2:** 106例患者的治疗反应[*n* (%)] Response to treatment of 106 patients [*n* (%)]

Index	The young group (*n*=21)	The young old group (*n*=70)	The old old group (*n*=15)	*P*
Objective response rate	7 (33.3)	37 (52.8)	8 (53.3)	0.284
Disease control rate	17 (81.0)	59 (84.3)	15 (100.0)	0.216
Best response				
Complete response	0 (0.0)	1 (1.4)	1 (6.7)	0.302
Partial response	7 (33.3)	36 (51.4)	7 (46.7)	0.351
Stable disease	10 (47.6)	22 (31.4)	7 (46.7)	0.138
Progressive disease	4 (19.0)	11 (15.7)	0 (0.0)	0.239

### 2.3 生存分析

在中青年组、年轻老年组和一般老年组患者中，中位随访时间分别为16.0个月(5.6个月-26.4个月)、11.9个月(7.3个月-16.5个月)和8.8个月(8.1个月-9.5个月)。分别有11例(52.4%)、43例(61.4%)和7例(46.7%)进展。中位PFS分别为9.1个月(95%CI: 2.0-16.1)、7.6个月(95%CI: 5.4-9.8)和10.9个月(95%CI: 5.0-16.8)(图 1)。依据临床特征分组的PFS进一步分析(表 3-表 5)，我们发现在年轻老年组中(表 4)，男性患者较女性患者PFS更长(13.5个月*vs* 5.1个月，*P* < 0.001)，PD-L1阳性患者较阴性患者PFS更长(14.7个月*vs* 7.5个月，*P*=0.011)，一线免疫治疗比二线及以上免疫治疗的患者的PFS更长(15.6个月*vs* 5.3个月，*P* < 0.001)。而在中青年组中(表 5)PS评分0分-1分的患者较2分患者PFS更长(18.8个月*vs* 2.8个月，*P*=0.005)，一线免疫治疗比二线及以上免疫治疗的患者的PFS更长(21.1个月*vs* 10.6个月，*P*=0.017)，以上差异均具有统计学意义。在一般老年患者组中(表 3)，各项临床特征与PFS差异均无统计学意义。因一般老年组中未发现与PFS相关的预后因素，且考虑到性别分组中女性患者占比过少及ECOG PS评分中2分的患者占比过少，因此在中青年组和年轻老年组中纳入病理类型、PD-L1表达、治疗方案和治疗线数等变量，构建多因素*Cox*比例风险模型(表 6)。结果发现仅在年轻老年组中，治疗线数对生存时间的影响具有统计学意义(HR=4.041, 95%CI: 1.950-8.376, *P* < 0.001)，接受一线免疫治疗的患者更获益。

**图 1:**
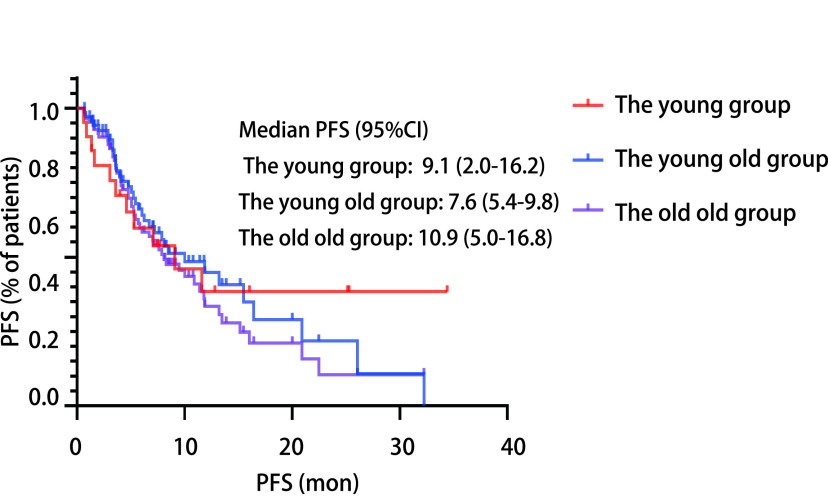
不同年龄组的PFS的*Kaplan*-*Meier*曲线比较 *Kaplan*-*Meier* curves of PFS compared with different age groups. PFS: progression-free survival.

**表 3:** 年龄≥75岁的患者不同临床特征与PFS之间的关系 Relationship between different clinical features with PFS in patients aged ≥75 yr

Factor	PFS (mon)	95%CI	*P*
Gender			0.095
Male	11.2	7.671-14.813	
Female	5.5	2.859-8.167	
Smoking status			0.128
Current/Former	11.2	7.671-14.813	
Never	5.8	3.377-8.310	
ECOG PS			0.965
0-1	10.5	6.637-14.263	
2	10.9	10.900-10.900	
Histological type			0.982
Squamous cell	8.5	5.961-11.118	
Non-squamous cell	10.2	5.382-14.918	
TNM stage			0.152
IV	10.5	9.059-12.011	
IIIb	8.4	4.347-12.406	
PD-L1 expression			0.096
Positive	12.1	8.194-16.095	
Negative	7.3	3.002-11.568	
Immunotherapy protocols			0.921
Monotherapy	10.4	3.625-17.140	
Combination therapy	9.1	7.016-11.156	
Number of treatment lines			0.498
First-line	12.6	7.746-17.539	
Others	8.5	6.054-10.894	

**表 4:** 年龄60岁-74岁的患者不同临床特征与PFS之间的关系 Relationship between different clinical features with PFS in patients aged 60 yr-74 yr

Factor	PFS (mon)	95%CI	*P*
Gender			< 0.001
Male	13.5	9.812-17.133	
Female	5.1	2.154-7.959	
Smoking status			0.014
Current/Former	13.1	9.371-16.803	
Never	6.9	3.432-10.439	
ECOG PS			0.458
0-1	11.2	8.324-14.007	
2	9.1	4.435-13.830	
Histological type			0.120
Squamous cell	11.2	8.538-13.891	
Non-squamous cell	10.1	6.686-13.597	
TNM stage			0.567
IV	11.4	8.813-14.622	
IIIb	10.4	6.574-14.258	
PD-L1 expression			0.011
Positive	14.7	10.284-19.016	
Negative	7.5	4.984-9.978	
Immunotherapy protocols			0.341
Monotherapy	13.4	7.727-19.073	
Combination therapy	9.9	7.298-12.510	
Number of treatment lines			< 0.001
First-line	15.6	11.228-20.026	
Others	5.3	3.267-7.339	

**表 5:** 年龄 < 60岁的患者不同临床特征与PFS之间的关系 Relationship between different clinical features with PFS in patients aged < 60 yr

Factor	PFS (mon)	95%CI	*P*
Gender			0.486
Male	17.8	10.209-25.324	
Female	6.6	0.000-16.430	
Smoking status			0.195
Current/Former	10.6	4.844-16.257	
Never	23.3	11.055-35.565	
ECOG PS			0.005
0-1	18.8	11.157-26.426	
2	2.8	0.508-5.045	
Histological type			0.736
Squamous cell	17.4	5.985-28.831	
Non-squamous cell	12.4	6.320-18.544	
TNM stage			0.893
IV	16.0	8.326-23.755	
IIIb	10.1	4.268-15.962	
PD-L1 expression			0.087
Positive	20.4	10.531-30.190	
Negative	9.0	2.533-15.546	
Immunotherapy protocols			0.055
Monotherapy	3.8	1.638-6.048	
Combination therapy	18.8	11.083-26.482	
Number of treatment lines			0.017
First-line	21.1	14.333-27.972	
Others	10.6	3.483-17.781	

**表 6:** 患者的PFS的多因素分析 Multivariate analyses of PFS in aged < 60 yr and 60 yr-74 yr groups

Factors	Aged < 60 yr (*n*=21)			Aged 60 yr-74 yr (*n*=70)	
	HR (95%CI)	*P*		HR (95%CI)	*P*
Histology (squamous cell *vs* non-squamous cell)	2.123 (0.398-11.334)	0.378		0.944 (0.445-2.001)	0.880
PD-L1 expression (positive *vs* negative)	3.137 (0.640-15.386)	0.159		1.868 (0.923-3.782)	0.083
Immunotherapy protocols (monotherapy *vs* combination therapy)	0.684 (0.137-3.418)	0.644		1.071 (0.474-2.421)	0.868
Number of treatment lines (first-line *vs* others)	8.071 (0.907-71.812)	0.061		4.041 (1.950-8.376)	< 0.001
HR: hazard ratio.					

### 2.4 安全性

在中青年组中共发生7例(33.3%)免疫相关不良反应(immune-related adverse event, irAE)，其中最常见的irAE是免疫相关性皮炎；在年轻老年组中共发生16例(22.9%)irAE，其中最常见的是免疫相关性肺炎；而在一般老年组中共发生3例(20.0%)irAE，其中最常见的是免疫相关性皮炎(表 7)。仅在年轻老年组中有6例患者发生高级别(≥3级)irAE：3例免疫相关性肺炎、1例免疫相关性肌炎、1例免疫相关性皮炎和1例免疫相关性肝炎。三组irAE发生率的差异无统计学意义(*P* > 0.05)。

**表 7:** 免疫治疗期间观察到的与免疫相关不良事件 Immune-related adverse events observed during immunotherapy

Factors	Aged < 60 yr (*n*=21)			Aged 60 yr-74 yr (*n*=70)			Aged≥70 yr (*n*=15)	
	Any grade	Grade≥3		Any grade	Grade≥3		Any grade	Grade≥3
Dysthyroidism	2 (9.5%)	0		5 (7.1%)	0		0	0
Pneumonia	0	0		5 (7.1%)	3 (4.3%)		1 (6.7%)	0
Arthritis	0	0		1 (1.4%)	0		0	0
Myositis	0	0		2 (2.9%)	1 (1.4%)		0	0
Dermatitis	3 (14.3%)	0		1 (1.4%)	1 (1.4%)		2 (13.3%)	0
Hepatitis	1 (4.8%)	0		2 (2.9%)	1 (1.4%)		0	0
Arrhythmia	1 (4.8%)	0		0	0		0	0
All	7 (33.3%)	0		16 (22.9%)	6 (8.6%)		3 (20.0%)	0

## 3 讨论

我们对北京胸科医院接受免疫治疗的NSCLC患者进行了回顾性分析，重点探讨了一般老年患者即年龄75岁及以上、年轻老年患者即年龄60岁-74岁和中青年患者即60岁以下患者接受免疫治疗的疗效比较。结果显示，三组患者接受免疫治疗后的ORR、DCR和PFS差异无统计学意义，在安全性方面，各组患者的irAE发生率差异均无统计学意义(*P* > 0.05)。

在亚组分析中，我们发现在年轻老年组和中青年组中，一线免疫治疗比二线及以上人群治疗的PFS都更长，在一般老年组中也能观察到类似的趋势。免疫治疗发挥疗效的机制与机体免疫状态密切相关，一线使用免疫治疗的患者人群通常免疫状态更佳，而先前接受过化疗的人群其免疫系统可能会受到破坏^[10]^。在年轻老年组中PD-L1阳性患者的PFS更长，在其余组中也能观察到这种趋势，尽管差异没有统计学意义。PD-L1表达水平是目前唯一被批准用于选择免疫治疗患者的标志物，这表明对于PD-L1阳性的NSCLC患者应积极使用免疫治疗。

最新的一些*meta*分析比较了接受免疫单药治疗的老年肺癌患者和年轻患者之间的疗效差异。Marur等^[11]^根据来自4项临床试验(CheckMate 057、KEYNOTE-010、OAK、POPLAR)的患者年龄评估总生存期(overall survival, OS)和存活率，纳入的都是先前经过治疗的NSCLC患者。在2, 824例患者中，21.5%的患者年龄 > 70岁，12%的患者年龄 > 75岁，对于 < 65岁、65岁-74岁和≥75岁的患者，中位OS分别为14.5个月、14.2个月和14.7个月。他们得出的结论是，对于65岁或以上的患者，ICIs的益处与年轻患者相似。Zhang等^[12]^同样评估了ICIs对老年肺癌患者的疗效。他们选择了涉及8, 176例患者的12项临床研究，其中46%(*n*=3, 730)的患者年龄至少为65岁。使用65岁作为截断年龄，与支持治疗组(standard of care, SoC)相比，ICIs在年轻组(HR=0.75, 95%CI: 0.65-0.87)和老年组(HR=0.81, 95%CI: 0.72-0.92)均改善了OS。然而，在基于75岁截断年龄下对老年组进行新的细分后，与65岁-75岁组相比，未能证明免疫疗法对75岁及以上患者有益处(HR=0.90, 95%CI: 0.64-1.25)。

同时不同国家的多项回顾性研究也得到了类似的结论。Grosjean等^[13]^比较了当地接受一线帕博利珠单抗治疗的晚期NSCLC的老年患者(年龄 > 70岁)和年轻患者有效性和安全性，他们发现老年患者和年轻患者的临床结果相似，ORR、中位OS和irAE发生率等均相似。Luciani等^[14]^对接受PD-1/PD-L1免疫疗法治疗的75岁以上NSCLC患者进行了多中心回顾性分析。总体中位PFS为5.6个月，中位OS为10.1个月，与参加临床试验的年轻患者中观察到的结果相似。

安全性是老年患者关注的另一个话题，与年龄相关的生理变化和合并症可能会增加与治疗相关的毒性并降低对治疗的耐受性^[15]^。在CheckMate 171研究中，除了老年人群中发生轻度腹泻的概率较高外，与年轻人群相比其他免疫相关不良事件发生率相似^[16]^。在CheckMate 153研究中，高级别治疗相关不良事件(treatment-related adverse event, TRAE)的发生率在所有亚组中相似，总体人群和老年人组的TRAE均为6%^[17]^。目前尚无关于ICIs联合化疗或ICIs联合抗血管的年龄特异性的安全性数据。

之前对于老年患者接受免疫治疗疗效差的考虑主要是因为他们的衰老与免疫系统退化相关，在接受治疗后会增加降低药物疗效或增加副作用的风险。免疫治疗的机制是通过恢复T细胞的功能来达到杀伤肿瘤的作用，而T细胞对病原体的反应随着年龄的增长而降低^[18]^。目前的研究认为对于老年患者来说年龄及PS评分不能完全地反映他们的身体状况，应根据指南建议进行老年综合评估(comprehensive geriatric assessment, CGA)以避免过度治疗或治疗不足^[19]^。因此需要在老年人群中进行更大规模的研究并评估他们的综合情况，才能得出更准确的结论。本研究由于是回顾性研究，不可避免地存在一定的选择偏倚。样本量偏小，在治疗方面选择的免疫药物不统一，治疗方案较多，但本研究的结果也更代表真实世界的临床实践。关于OS的评估由于随访时间的问题数据不够成熟，我们将继续关注。

综上所述，本研究通过对真实世界的数据分析，发现老年患者和年轻患者接受免疫治疗后的疗效相近，不良反应发生率也相近。说明年龄不是临床医生对于老年NSCLC患者是否使用免疫治疗的主要考虑因素，应当综合患者的临床特征，包括PS评分、肿瘤因素、病理因素、基因突变状态等，在患者能够耐受的前提下积极使用免疫治疗。此外，未来的研究需进一步探讨如何对老年患者进行科学合理的免疫状况评估，同时，有必要开展前瞻性老年患者的临床试验，探索适合老年患者的免疫治疗方案、用药剂量及护理方案。
